# Narrative review of methodological advances in human milk fortification: for better preterm infant growth

**DOI:** 10.3389/fped.2024.1466528

**Published:** 2024-11-28

**Authors:** Jiabin Yang, Qin Tang, Ping Zhou

**Affiliations:** ^1^ Graduate School of Shantou University Medical College, Shantou, China; ^2^ Department of Pediartics, Longgang Central Hospital, Shenzhen, China; ^3^ Department of Neonatology, Baoan Women’s and Children’s Hospital, Shenzhen, China

**Keywords:** preterm infants, human milk fortifier, standard fortification, individualized fortification, extrauterine growth retardation

## Abstract

It is generally suggested that human milk alone might not be enough to meet the nutritional requirements of very preterm infants, necessitating the use of nutritional fortification. The composition of human milk varies among individuals and changes over time, challenging the assumption that protein content and energy density remain constant during standard fortification. Consequently, it has led to suboptimal body growth rates in most very preterm infants compared to fetuses of the same gestational age. In light of this, personalized fortification and innovative fortification strategies have been introduced. This paper aims to review the importance of fortification and the shortcomings of standard fortification, as well as describe and evaluate the advantages and limitations of various individualized fortifications. The optimal use of human milk fortification, in accordance with the nutrient content of human milk and the physiological maturity and growth of preterm infants, is a crucial aspect of the field of preterm infant nutrition.

## 1 Introduction

In 2020, there were 13.4 million preterm births globally, representing 9.9% of all newborns. Approximately 15% of these infants were very preterm (1). With the advances in perinatal medicine and neonatal intensive care, the survival rate of very preterm infants has increased, while the increasing complication rate has become one of the major public health issues (2). Adequate and balanced nutrition is the material foundation for the healthy growth of preterm infants and one of the key links to improving their survival rate, which is related to their near-term growth and disease regression and directly affects their long-term prognosis (3).

Mother's own milk (MOM) is the optimal choice for feeding very preterm infants, as it significantly reduces all-cause mortality and the incidence of multiple complications in very preterm infants and improves long-term neurodevelopment and cardiovascular health (4, 5). However, the protein-energy ratio (P/E) of unfortified human milk is approximately 1.8 g/100 kcal, which is considerably lower than the recommended value of 3.2–4.1 g/100 kcal to support optimal protein utilization and body growth in preterm infants (6, 7). When MOM is insufficient or lacking, human donor milk (HDM) is frequently selected as a supplement or substitute. HDM, which is derived primarily from mothers of term infants a few months after birth, has lower protein content and calories (8). This nutritional deficiency can result in extrauterine growth retardation (EUGR) in preterm infants, posing the risk of neurocognitive impairment and other adverse health outcomes (3, 7–9). Therefore, the incorporation of human milk fortifier (HMF) into MOM or HDM to facilitate the growth and development of premature infants has become a widely acknowledged and standardized practice in clinical settings (10, 11).

Methodologically, standard fortification is commonly employed in clinical practice (11). However, very preterm infants fed with standard fortification still have a high incidence of EUGR. Consequently, there has been a great deal of research into how to optimize human milk fortification. The objective of this paper is to conduct a systematic review of the methodology of fortification and summarize the strengths and weaknesses of standard and individualized fortification in order to improve the understanding of human milk fortification in preterm infants.

## 2 Standard fortification

Standard fortification (SF) is the most prevalent method of human milk fortification in clinical practice (11). Instead of targeting the actual needs of the infant, this method assumes that human milk has a consistent protein content and energy density, and adds a fixed dose of a multi-component fortifier to a certain amount of human milk (10). It is typically administered when enteral feeding reaches 50–100 ml/kg/day and is initially half fortified, increasing to full fortification within 3–5 days if tolerated (11). SF offers several advantages, including ease of application and simplicity of administration. The Cochrane systematic review (12) demonstrated that, in comparison to unfortified human milk, SF facilitated weight, length, and head circumference growth during hospitalization in preterm infants. However, no significant differences were observed in long-term growth and neurodevelopment.

The most significant limitation of SF is that it fails to account for fluctuations in human milk composition throughout lactation, and does not adapt to changes in human milk composition and differences in the individual requirements of preterm infants. The protein content of human milk can decline from 1.4–1.5 g/dl in the initial two weeks of lactation to 1 g/dl in weeks four to six and the composition of human milk varies considerably between mothers (13, 14). Consequently, SF does not provide sufficient protein for preterm infants (15). It was found (16) that the caloric intake was adequate after SF, whereas the actual protein intake of 2.8–2.9 g/kg/day and the protein-energy ratio of 2.2–2.7 g/100 kcal were lower than those recommended by the ESPGHAN (8). Maly et al. demonstrated that the majority of preterm infants who received SF were unable to meet the recommended protein intake, and their growth lagged behind that of intrauterine infants of the same gestational age (14). A study found that 58% of very preterm infants after SF had EUGR at discharge (17), and a similar finding was observed in a Chinese multicenter survey (18), in which the incidence of EUGR in extremely preterm infants was as high as 81%. Furthermore, the protein content of different brands of fortifier results in a significant difference in the amount of human milk protein added (1.0–1.7 g/dl) after SF, which may lead to disparate growth outcomes (3). SF with 1 g or 1.42 g of protein per 100 ml of human milk was found to result in inadequate protein supply in preterm infants in up to 95% and 74% of cases, respectively (19).

In light of these limitations, research has sought to introduce fortification at an earlier stage to facilitate growth and mitigate the risk of EUGR. Shah et al. observed that early fortification (enteral feeding reached 20 ml/kg/days) enhanced protein intake without triggering an increase in adverse events (20). Ginovart also documented that early fortification enhanced head circumference and weight gain in hospitalized preterm infants (21). In another study, fortification was initiated at the time of the first enteral feeding, but it did not improve growth in the first four weeks of life compared with late fortification (22). The results of recent meta-analyses suggest that early fortification has little or no effect on body growth during hospitalization in very preterm infants (23). Hence, the available evidence is insufficient to support or negate early fortification. Further large trials are needed to provide sufficiently high-quality and accurate data to inform clinical practice.

## 3 Hyper-dose fortification

Hyper-dose fortification (HF), also named intensive or aggressive fortification, is the addition of a greater quantity of fortifier than the standard dose in human milk, or a standard dose of fortifier to a smaller amount of human milk, with the aim of increasing nutrient delivery. This method is primarily employed in preterm infants who have poor growth after standard fortified feeding and where individualized fortification is not available. Typically, the dose of fortification is increased to 1.25 times the standard dose, and if growth remains inadequate, the dose can be increased to 1.5 times. The use of higher concentrations of HMF has been described particularly with DBM feedings and with fortification using HM-HMF (24, 25). Kanmaz et al. (26) observed that preterm infants who received moderate (1.2 times the standard dose) or intensive fortification (1.5 times the standard dose) were larger in head circumference compared to those who received SF. No significant differences were observed in weight, length, and laboratory indices such as urea nitrogen, calcium, phosphorus, and clinical outcomes. This indicates that HF, in addition to increasing head circumference, did not enhance other short-term growth outcomes or BUN levels reflecting greater protein intake.

Although HF may be an acceptable approach for preterm infants with poor growth who are unable to implement individualized fortification, the main problem with standard fortification is that protein is inadequate while calories are adequate (11). Therefore, HF carries the risk of excess energy intake while supplementing with more protein, and the protein-energy ratio remains below the recommended level, which may lead to a risk of metabolic disease in preterm infants with a high body fat composition in the long term (27). Another concern is that the electrolytes and micronutrients in the fortifier are formulated based on standard fortification, and hyper-dose fortification has the risk of leading to excessive electrolyte and micronutrient intake. While Kanmaz's study did not find a difference in calcium and phosphorus levels, it lacked important blood sodium and potassium data (26). The osmolality threshold for enteral feeding is generally no more than 500 mOsm/kg, although the recently published guidelines do not clearly define it (28). It is possible that HF may result in the hyperosmolarity of human milk (29), particularly if the fortifier used contains high levels of deeply hydrolyzed proteins and carbohydrates (30).

For these reasons, the use of HF has been reported in few studies, and none of the nutritional guidelines recommend this approach. In light of the aforementioned risk, any clinical use of this approach must be carefully monitored for potential adverse effects.

## 4 Individualized fortification

Due to the limitations of SF and the potential risks associated with HF, individualized fortification has been implemented in some neonatal intensive care units (NICUs). It is regarded as a solution to the problem of protein malnutrition following SF and is currently recommended by international guidelines (11, 28). Individualized fortification is based on two main methods: adjustable fortification and targeted fortification. The objective is to achieve the target nutrient intake for preterm infants of the appropriate gestational age.

### 4.1 Adjustable fortification

The concept of adjustable fortification (AF) was first proposed by Moro et al. (31) and developed by Arslanoglu et al. (32) in a standardized form. The approach is based on the metabolic response of preterm infants, when renal function and access are normal and protein intake is closely related to blood urea nitrogen (BUN) levels (33). BUN was measured twice weekly following the commencement of standard fortification to assess protein metabolism. SF was continued if the BUN level was between 10 and 16 mg/dl, with an additional 0.4 grams of protein fortifier based on SF if BUN was <10 mg/dl, and 0.8 grams of protein if BUN remained <10 mg/dl, up to a maximum of 1.2 g. Conversely, if BUN > 16 mg/dl, the fortification was gradually reduced to a minimum of 1/4 standard fortification (32). The AF method does not necessitate an analysis of human milk composition and is easy to apply clinically. It increases protein intake without increasing total energy or fluid volume, is well tolerated, and improves physical growth and the long-term prognosis of preterm infants during hospitalization (34, 35). Furthermore, it also increases the rate of physical growth after discharge and may also improve long-term neurodevelopmental outcomes (36). Ergenekon et al. demonstrated that infants receiving AF exhibited significantly higher indices of psychosocial and psychomotor development (BSID-III scores) than those in the SF group at postmenstrual age 18 months (37), as well as significantly higher hearing and speech scores (38).

The use of BUN as an indicator of protein metabolic response is a feature of adjustable fortification. However, BUN does not always fully reflect protein intake, particularly in very preterm infants with a high catabolic rate in the first few weeks of life. In such cases, urea levels may be elevated regardless of protein intake or renal function (39). Furthermore, the limited capacity for urea synthesis and/or renal excretion in preterm infants, as well as increased catabolism in some disease states, do not accurately reflect protein metabolism (40). The clinical study has demonstrated that greater protein intake does not necessarily result in an elevated BUN level. Consequently, BUN is not considered a reliable indicator of adequate protein intake (26). Furthermore, BUN measurement necessitates repeated invasive procedures, which result in increased patient discomfort, and the frequent blood collection and analysis also impose a greater burden on healthcare workers.

To address these shortcomings, a non-invasive AF has been proposed. Mathes et al. (41) demonstrated a high positive correlation between plasma BUN concentration and urine urea-creatinine ratio as well as actual protein intake in preterm infants. It is therefore proposed that the use of the urine urea-creatinine ratio as a valid indicator of protein metabolism could help to estimate actual protein consumption in preterm infants.

### 4.2 Targeted fortification

The protein content of human milk typically declines with the progression of lactation in a mother and may also exhibit significant inter-individual variability (13–15). Therefore, target fortification (TF) is achieved by analyzing human milk composition at regular intervals (daily or twice weekly) to obtain measured macronutrient component data, and targeting guideline-recommended intakes by adding additional proteins, fats, or carbohydrates to standard fortified human milk to meet the nutritional needs of preterm infants (42, 43). The analysis time is brief, and the results are highly accurate, rendering it suitable for use in NICUs.

TF addresses the variability of macronutrients in breast milk through real-time measurement and supplementation, thereby optimizing the P/E ratio and preventing protein deficiency in infants, while ensuring better growth quality. TF has been demonstrated to improve nutrient intake and the quality of growth, including length, head circumference, fat, and fat-free mass, compared with SF (42–46). Furthermore, the osmolality of human milk following TF was within safe limits, and no signs of gastrointestinal or metabolic intolerance were observed (44).

The use of human milk analyzers and ultrasonic homogenizers is required for TF, which are relatively complex pieces of equipment and require frequent sampling and analysis as well as frequent calibration of the analyzers. This increases the workload in the NICU by 10–15 min per patient per day, with high instrumentation and labor costs that are not readily available for widespread use in NICUs (11, 42). Labor costs have been reduced by reducing the frequency of testing, for example, by switching to weekly analysis, with macronutrient intake remaining within ±5% of target levels (47, 48). A recent survey revealed that only 10% of NICUs were able to perform human milk analysis for clinical purpose, while adjustable fortification was practiced in 41.3% (49). It is crucial to acknowledge that TF does not account for individual metabolic and absorptive differences in preterm infants, potentially leading to under- or over-intake of nutrients. Furthermore, the accuracy of human milk analysis methods and the inaccessibility of various single component fortifiers in resource-limited countries represent additional challenges (50–52).

### 4.3 Comparison of adjustable and targeted fortification

Studies have demonstrated that either adjustable or targeted fortification is more effective than SF in promoting early physical growth in preterm infants (10). However, there is a paucity of literature comparing the two approaches, and the results are inconsistent. Kadıoğlu et al. (53) found that the rate of growth in length and head circumference in the TF group was lower than that in the AF group. Conversely, the study by Bulut et al. found that the rate of growth in weight and head circumference in the TF group was significantly better than that in the AF group (54). Only one study compared the incidence of bronchopulmonary dysplasia and metabolic bone disease in preterm infants receiving targeted vs. adjustable fortification. Neither difference was statistically significant (53). A meta-analysis of these two studies was not quantitatively combined due to the limited number of studies and cases within each, and the observed effect remains to be further confirmed (55).

## 5 New strategies of fortification

Given the disadvantages of adjustable and targeted fortification, researchers are also attempting to refine them or develop innovative strategies of fortification.

### 5.1 Adapted fortification

Non-invasive targeted fortification is more aligned with the optimal needs of very preterm infants and represents a promising approach. However, it necessitates the use of specialized equipment and is time-consuming, rendering it impractical in most NICUs (49). Consequently, a practical fortification strategy to optimize protein intake without the necessity for human milk analysis has been proposed: adapted fortification.

Minarski et al. (19) developed an equation for calculating human milk protein content: protein [g/100 ml] = 6.755/postnatal days + 0.852. This was achieved by analyzing the protein composition of human milk from 457 samples taken from 41 mothers during different stages of lactation and performing regression analyses and mathematical modeling of the trend towards a decrease in protein content with increasing lactation days. A validation cohort of 10 mothers with 141 human milk analyses was used to validate the equation, which was followed by target-volume protein supplementation, and the results demonstrated that the target protein supply was achieved in more than 95% of human milk samples. This straightforward, non-invasive fortification is regarded as a modified form of TF. The method compensates for changes in human milk protein content with minimal additional effort and establishes a viable fortification strategy for daily practice in NICUs.

### 5.2 Stepwise corrected fortification

Stepwise corrected fortification (SCF) is also a practical method based on the same fact that the protein in human milk decreases progressively at different stages of lactation. Started with standard fortification, and then progressively increased adding the protein and calories in human milk at different weeks after the birth of preterm infants in order to reach the recommended intake (56).

Pillai et al. (56) observed that the protein content of human milk declines in early lactation and is lower than expected from week 3 onwards. Consequently, standard fortification was introduced for the first 2 weeks of life, followed by an additional 1.27 g of protein fortifier for weeks 3–4 of life, and then 1.57 g of protein for weeks 5–6 of life, and 1.81 g of protein at week 7 of life and beyond. Since DHM was lower in protein and calories, 2.05 g of protein were added. Following the aforementioned fortification procedure, 68% of human milk samples fell within the recommended range for protein and calories, in comparison to only 5% in all human milk samples fortified with SF and 0% in samples after five weeks. It was observed that SCF had a greater impact on protein content compared with calories. Thus, SCF markedly augmented the final protein and calorie content of human milk, thereby offering a promising avenue for enhancing nutritional intake in preterm infants.

### 5.3 Human milk calorie guide

In the course of the study of SCF, Pillai et al. observed that the degree of yellow coloration of human milk may be correlated with the measured calories. Consequently, they introduced the bedside color tool, the Human Milk Calorie Guide (HMCG) (57). The objective of this tool is to predict the calories in human milk in resource-limited settings where human milk analyzers are not available.

The color tool comprises nine colors, divided into three rows. The lighter “watery” shade is found in row A, the “normal white” shade in row B, and the “creamy yellow” shade in row C. Each row contains three colors, with the lighter shades located on the left side and the darker shades on the right side. It was assumed that the calorie values of human milk would increase in line with the “yellowness” of the samples, from those in row A to those in row C. The MOM and DHM samples were then color-matched and analyzed for macronutrients. Among DHM samples, the color tool demonstrated the greatest accuracy in predicting low calorie values (<55 kcal/dl, AUC of 0.87 for row A), exhibited average accuracy in predicting high calorie values above 70 kcal/dl (AUC of 0.77 for row C), and exhibited poor prediction performance for MOM (56). The authors concluded that this color tool is a reliable predictor of the lower caloric range of donor milk and has the potential to improve donor milk fortification practices.

### 5.4 Breast milk protein percentiles (BMPs)

Arıkan et al. (58) analyzed the protein content of weekly human milk from 108 mothers of preterm infants in four groups of extremely preterm, very preterm, early preterm, and mid-late preterm during the first five weeks postnatal. The P10, P25, P50, P75, and P90 percentile curves of changes in human milk protein content of preterm infants of different gestational ages and postnatal weeks of age were plotted to create a new practical and individualized fortification guideline, replacing the laborious targeted or adjustable fortification currently in use.

The objective of these novel fortification above mentioned is to address the limitations of standard and adjustable fortification or targeted fortification in providing adequate nutrition for preterm infants, based on diverse clinical settings and different resource accessibility. However, the efficacy and safety of these methods remain unproven, as there is a paucity of clinical applications or comparative studies. It is also worth considering whether these equations or models derived from human milk composition data from a particular population are suitable for use in other populations from different countries and regions. The principle, advantages and disadvantages, and current state of application of the most of fortification methods presented are summarized in Table 1.

**Table 1:** Characteristics of different human milk fortification methods.

Method	Principle	Advantages	Disadvantages	State of application
Standard fortification (10) (SF)	Addition of a fixed amount of HMF to per 100 ml of HM to reach an assumed macronutrient composition.	Practical and easy-to-use	Assuming a fixed protein content for all milk without considering intra-, inter-individual and temporal variations. Despite SF, many very preterm infants continue to have suboptimal growth.	Well-researched and most widely used based on international guidelines (11, 28, 49, 59).
Hyper-dose fortification (26)	1.25 × SF or 1.5 × SF	Increase protein intake	Risk of excessive energy intake and high osmolality. Difficulty in achieving the recommended protein-energy ratio.	Little researched, a compromise practice when individualized fortification is unavilable in preterm infants with undernutrition (26).
Adjustable fortification (31, 32)	Protein adequacy is monitored by BUN twice weekly with a cut-off value 3.5–5.7 mmol/L. If the level is <3.5 mmol/L, a different level of extra protein is added to the SF fortification.	Practical and not labor-intensive. Doesn't need expensive devices. Taking into consideration each infant's protein requirement.	Twice-weekly blood draws with risk of pain and medical anemia.	Well-researched and used in some NICUs based on international guidelines (11, 28, 48, 49, 59).
Targeted Fortification (43–48)	Macronutrients in HM are analyzed, and based on the results, human milk is supplemented with single-nutrient fortifiers.	Nutrient intakes can meet the recommended value of the feeding guidelines. All macronutrients can be supplemented.	Expensive devices and labor intensive, not widely applicable. Failure to account for individual differences in nutrient absorption and metabolism in preterm infants.	Well-researched and used in few NICUs based on international guidelines (11, 28, 43–49, 59).
Adapted Protein Supplementation (19)	No need for human milk composition analysis. Targeted protein supplementation based on calculated HM protein values obtained from a validated equation.	Takes into account the variability of human milk over the lactation stage. No need for expensive devices and repeated human milk composition analysis. Highly practical.	The equation can only guide protein addition supplementation. 5% chance of over- or undernutrition of protein. The equation is derived from human milk data from a single region, and its general applicability is questionable.	Novel method. Still being evaluated and need more validation. Not included in guidelines (19, 49).
Stepwise corrected fortification (56)	Started with SF and then increased adding the protein and calories in human milk at different weeks after birth, based on the protein in human milk decreasing over time.	Takes into account the variability of human milk composition. Some practical.	Complicated addition method. More than 30% still fail to meet recommended protein and calorie intakes.	Little researched. Not included in guidelines (56).

BUN, blood urea nitrogen; HM, human milk; HMF, human milk fortifier; NICU, neonatal intensive care unit; SF, standard fortification.

## 6 Conclusions and outlook

In conclusion, research has shown that adding a fortifier to human milk can improve the nutritional status and growth rates of premature babies. According to the data, TF or AF might be a more effective fortification technique than SF. However, the optimal individualized fortification strategy has yet to be determined. Therefore, more research is needed to assess the safety of the different approaches in relation to significant clinical outcomes, such as death, necrotizing enterocolitis, bronchopulmonary dysplasia, sepsis, neurological outcomes, and growth in very preterm infants after NICU discharge.

There could not be a perfect fortification that fully meets the nutritional needs of preterm infants without exacerbating adverse effects. This is due to the fact that it requires not only fortification method but also the fortifier type, timing, and other factors. In any event, clinical research on individualized fortification is one of priority for future nutritional care of preterm infants due to the diversity of human milk content and metabolic alterations in these babies. This is to make sure that preterm infant's nutritional intake satisfies the recommended levels, enhancing both their short-term growth and long-term outcomes.
